# Comparison of Soil Microbial Composition in Rhizospheres Between Wilt Disease-Resistant and Susceptible Melon Varieties

**DOI:** 10.3390/microorganisms13020444

**Published:** 2025-02-18

**Authors:** Lulu Qiu, Yu Zhu, Xinni Li, Yuchen Qin, Guifen Li, Yunfeng Ye, Yi He, Jinyan Huang, Shangdong Yang

**Affiliations:** 1 Guangxi Key Laboratory of Agro-Environment and Agro-Products Safety, National Demonstration Center for Experimental Plant Science Education Guangxi Agricultural College, Guangxi University, Nanning 530004, China; 18778409037@163.com (L.Q.); 15660553993@163.com (Y.Z.); lixinni19@163.com (X.L.); 18176718793@163.com (Y.Q.); 2 Horticultural Research Institute, Guangxi Academy of Agricultural Sciences, Nanning 530007, China; liguifen79@163.com (G.L.); yeyunfeng111@126.com (Y.Y.); gxnkyhy@163.com (Y.H.)

**Keywords:** melon, wilt resistance, high-throughput sequencing, root system, rhizosphere microorganisms

## Abstract

To screen out the bio-control soil microorganisms for preventing melon wilt, soil microbial compositions in rhizospheres between wilt-resistant and susceptible melon varieties were analyzed. The results showed that the soil fungal richness in rhizospheres of wilt-resistant melon varieties (MT) was significantly higher than that of wilt-susceptible melon varieties (MS). Additionally, in comparison with MS, soil bacterial compositions, such as Proteobacteria, Bacteroidota, *Acidibacter, Streptomyces*, etc., and the soil fungal compositions, such as *Penicillium*, *Derxomyces*, *Aspergillus*, and *Talaromyces*, enriched; also, *Trichoderma*, *Gibellulopsis*, and *Pseudallescheria* decreased in rhizospheres of wilt-resistant melon varieties (MT). Moreover, *Mycothermus*, *Zopfiella*, and *Cladorrhinum* were the unique soil-dominant fungal genera in rhizospheres of MT. All the above results suggested that the soil bacterial communities, such as Proteobacteria, Bacteroidota, Acidibacter, *Streptomyces*, etc., and the soil fungal communities, such as *Penicillium*, *Derxomyces*, *Aspergillus*, *Talaromyces Mycothermus*, *Zopfiella*, and *Cladorrhinum*, could be speculated as the potential soil bio-control microorganisms for preventing melon wilt.

## 1. Introduction

Melon (*Cucumis melon* L.) has a high nutritional value, its fruit is sweet and rich in sugar, starch, and a small amount of protein, minerals, and other vitamins, and it is widely cultivated around the world. Particularly, China is the largest production country in the world. According to the Food and Agriculture Organization of the United Nations database, the total production of melons in China exceeds 1.42 million tons in 2022, which was accounting for about 50% of the world’s total production (https://www.fao.org/faostat/zh/#compare, accessed on 8 July 2023). However, Long-term large-scale cultivation of melons inevitably exacerbates the outbreak of soil-borne diseases. When grown in the same field without rotation, melons are highly susceptible to *Fusarium oxysporum* f. sp. *melonis* [1]. The Fusarium wilt disease caused by this pathogen leads to melon death [2], severely impacting its yield and quality [3]. Therefore, farmers have tried to mitigate the occurrence of wilt disease by selecting wilt-resistant melon varieties [4], rotation [5], chemical [6] or physical [7] controls, and microbial fertilizers [8] for prevention. However, traditional cultivation methods, as well as physical means such as sun-drying and composting, often have the drawbacks of long cycles and limited effectiveness [9,10]. Therefore, farmers tend to prefer chemical control methods that yield quick results. Although chemical control can achieve its target in the short term, long-term application of pesticides and chemical fertilizers not only will induce soil pollution, but also lead to serious damage to the soil environment [11,12] and drug resistance of the pathogenic bacteria [2], i.e., it invariably increases production and ecological costs. Additionally, the pathogen of Fusarium wilt in melons can survive in the soil for a long time and infect crop roots, making it difficult to control with fungicide sprays. In conclusion, selecting disease-resistant varieties and using biological control methods are very effective and valuable approaches for controlling Fusarium wilt in melons [13,14,15].

The use of microbial strains is considered a non-toxic and genetically stable biological control strategy, showing effectiveness against various plant pathogens even at low concentrations [16]. Many antagonistic strains have been proven to be effective biological control agents under controlled laboratory or greenhouse conditions [17,18,19,20]. It is well known that soil microorganisms in rhizospheres are closely associated with plant production [21]. The root system attracts soil microorganisms around the rhizosphere area through secretions, forming a unique rhizosphere soil microbial community to meet its functional needs, and also regulates the sequential expression of genes related to biocontrol, which is widely recognized as the “cry for help” theory. In addition, plants also regulate the sequential expression of genes related to biocontrol and plant growth promotion in microorganisms through the sequential expression of root exudates across plant developmental stages [22]. Rhizosphere microorganisms can be divided into two categories, i.e., beneficial and harmful bacteria; among them, beneficial bacteria can promote nutrient cycling [23,24], decompose root secretions [25], produce phytohormones [26], and antagonize pathogens [27] to create a favorable environmental condition. Conversely, harmful bacteria can impede plant normal growth and development through toxin production, nutrient competition, and invasion of the plant [28]. Rhizosphere microorganisms play an important role in regulating soil microecological balance and promoting healthy plants [29,30]. This is because the rhizosphere microbial community is shaped by plant genotypes, soil types, plant fertility periods, and external climatic conditions [31,32,33].

Previous studies have confirmed that disease-resistant crop varieties, such as tomato [34], barley [31], and sugarcane [35], accumulate more microorganisms that enhance plant disease resistance in the rhizosphere and within the plants compared to susceptible varieties. Particularly, the enrichment of beneficial flora in disease-resistant crop varieties not only could enhance nutrient uptake, but also could reduce the harm of pathogenic bacteria. Melon varieties also showed significantly different resistances to wilt, which can be classified into wilt-resistant and susceptible melon varieties [36]. Compared to wilt-susceptible melon varieties, wilt-resistant melon varieties possess more resistance genes [37,38], higher activity of various resistance enzymes [39,40], and thicker cell wall structures or more developed duct systems [41,42], all of which are beneficial for resisting Fusarium wilt. The rhizosphere of plant varieties can selectively recruit a large number of disease resistance-related microorganisms, which are important biocontrol microbial resources for inhibiting pathogens [43].

Therefore, our study investigates the soil microbial community structure in rhizospheres of different Fusarium wilt-resistant melon varieties for screening out antagonistic microbes and elucidating the disease resistance mechanisms of resistant melon varieties.

## 2. Materials and Methods

### 2.1. Field Site Description and Experimental Designs

This experiment was conducted at the experimental base of the Horticultural Research Institute of Guangxi Academy of Agricultural Sciences (22°46′ N, 108°10′ E). The soils at the experimental base were red loam with pH 5.31, organic matter content 13.9 g·kg^−1^, and the total contents of nitrogen, phosphorus, and potassium were 0.81, 0.39, and 4.68 g·kg^−1^, respectively. Meanwhile, the contents of available nitrogen, phosphorus, and potassium were 53.71, 20.12, and 82.34 mg·kg^−1^, respectively. The melon varieties were all provided by the Institute of Horticulture, Guangxi Academy of Agricultural Sciences, i.e., three fusarium wilt-resistant melon varieties (Shan tian 1, Qiang shi, 985; abbreviated MT group) and three fusarium wilt-susceptible melon varieties (Hui yu, Qiaoyu, Chengmi, abbreviated MS group) were used in this experiment. All melon varieties were planted in the same field and grown under the same management.

### 2.2. Soil Samples Collection

Sampling was conducted on 19 May 2023, plants were randomly selected in the experimental field, using the shaking-off method as described by Riley and Barber [44], i.e., a sterilized shovel was used for loosing soil around the plant roots (depth of about 30 cm and 40 cm diameter). And then the whole plant was pulled up, shaking off the adhering soil, and taken back to the laboratory. Meanwhile, soil samples without melons were also collected for the background (CK). Soil samples were sieved through a 2 mm stainless-steel mesh and stored in a refrigerator at 4 °C for immediate analysis or at −80 °C for later analyses.

### 2.3. Test Methods

#### 2.3.1. Analysis of Soil Microbial Diversity

Total DNA was extracted according to the instructions of the E.Z.N.A. DNA Kit (Omega Company, Norwalk, CT, USA). DNA concentration and purity were detected by a NanoDrop 2000 spectrophotometer (Thermo Company, Waltham, NJ, USA), and the purity and quality of the genomic DNA were checked on 1% agositol gel. The ABI GeneAmp^®^ type 9700 (ABI, Carlsbad, CA, USA) was used for the PCR, and the products were recovered by 2% agarose gel electrophoresis and purified by an AxyPrep. Their specific primers and sequence types are shown in Table 1.

Illumina Miseq sequencing: PCR products from the same samples were recovered by product purification using the AxyPrep DNA Gel Extraction Kit (Axygen Biosciences, Union City, CA, USA), mixed and assayed for recovery on a 2% agarose gel, and quantified by quantifying the recovered products using a Quantus™ Fluorometer (Promega, Madison, WI, USA). According to the Illumina MiSeq platform (Illumina, San Diego, CA, USA) standard operating procedure, the purified amplified fragments were constructed into a library.

#### 2.3.2. Statistical Analyses

Bacterial and fungal community composition and functional prediction analyses were carried out by the I-sanger cloud data analysis platform (Majorbio Bio-Pharm Technology Co., Ltd., Shanghai, China), and the experimental data were statistically organized using Excel 2021 and SPSS 27.0. The significance analyses were conducted using the Wilcoxon rank sum test, with microbial diversity and richness expressed as Shannon and Simpson, Ace, and Chao1 indices, respectively. The results were all expressed as the mean ± standard deviation.

## 3. Results

Raw data from sequencing of rhizospheres bacteria and fungi of melon have been deposited in the NCBI database under accession numbers PRJNA1088444 and PRJNA1088463, respectively.

### 3.1. Soil Microbial Diversity in Rhizospheres Between Wilt-Resistant and Susceptible Melon Varieties

As shown in Table 2, the coverages of soil microbial communities of CK, MS, and MT were all above 98.0%, which indicated that the sequencing results could represent the real situation. In comparison with the wilt-susceptible melon varieties (MS), not only the soil bacterial diversity, but also soil bacterial richness in rhizospheres of wilt-resistant melon varieties (MT) were not significantly different from MS. However, although soil fungal diversity was not significantly different, the soil fungal richness in the rhizospheres of MT was significantly higher than that of MS.

**Figure 1:**
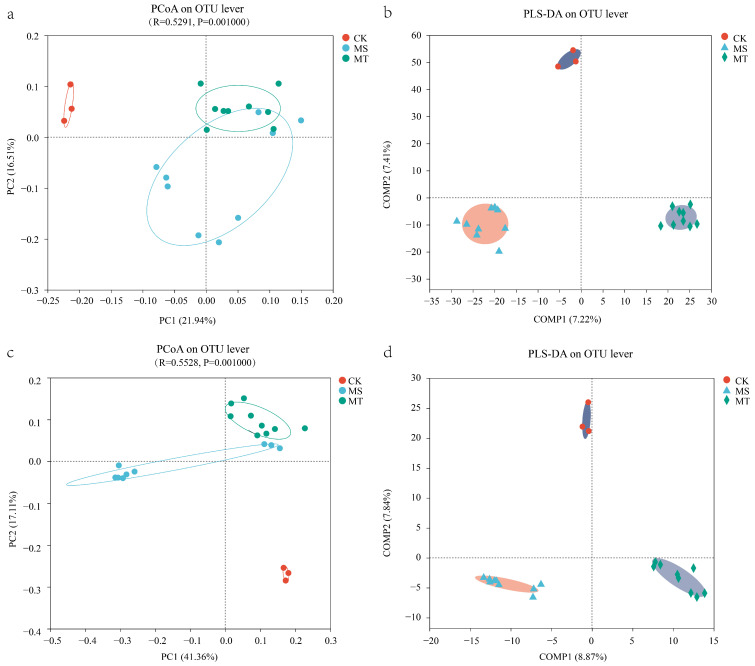
Comparison of soil microbial communities in rhizospheres between MT and MS melon varieties. (**a**) PCoA of rhizosphere bacterial communities at the OTU level. (**b**) PLS-DA score plot of rhizosphere bacterial communities. (**c**) PCoA of rhizosphere fungal communities at the OTU level. (**d**) PLS-DA score plot of rhizosphere fungal communities.

At the OTU level, the PCoA (Principal Coordinates Analysis) results revealed that the total variations of soil bacterial (Figure 1a) and fungal (Figure 1c) compositions in rhizospheres of MS, MT, and CK were 38.45% and 58.47%, respectively. Meanwhile, the similarity of soil bacterial and fungal communities in rhizospheres of different resistant melon varieties was also assessed by PLS-DA (Partial Least Squares Discriminant Analysis). And the results showed that soil bacterial communities in rhizospheres of melons (MT and MS) and background soil (CK) were distinctly clustered, respectively (Figure 1b). This result indicates that the composition of soil bacterial communities in the rhizospheres of MS, MT, and CK are significantly different. Viz., different soil bacteria were recruited in rhizospheres by various melon varieties with different resistance to Fusarium. In addition, soil fungal communities also showed a similar trend in rhizospheres of different melon varieties with soil bacteria (Figure 1d).

### 3.2. Soil Microbial Compositions in Rhizospheres Between Wilt-Resistant and Susceptible Melon Varieties

**Figure 2:**
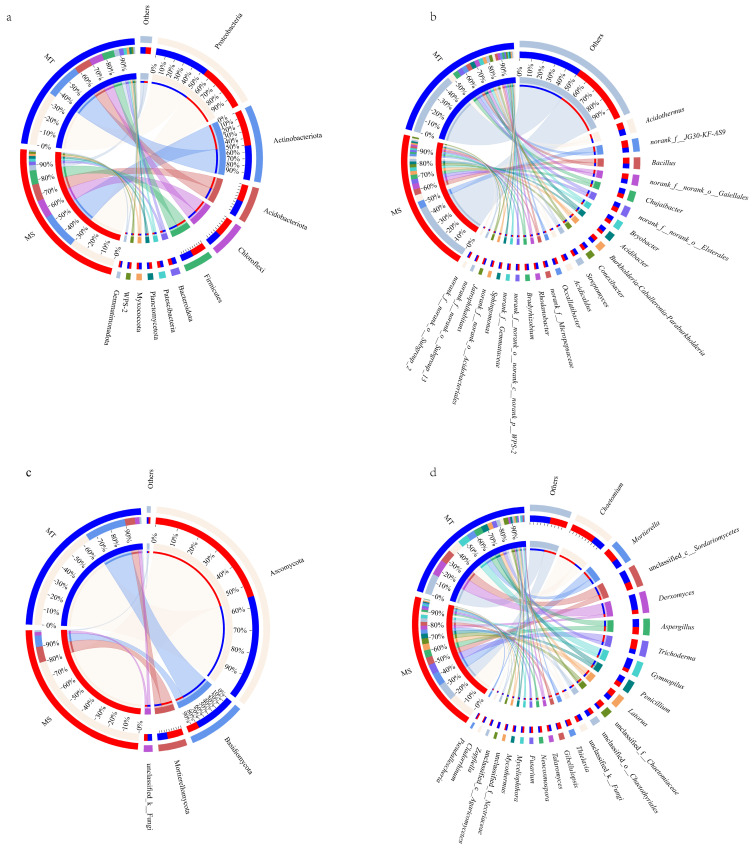
Distributions of rhizosphere bacteria (**a**,**b**) and fungi (**c**,**d**) between MT and MS varieties at the phylum and genus levels.

At the phylum level, 11 dominant soil bacterial phyla (>1% abundance share, below) could be detected in rhizospheres of MS and MT. Among them, Proteobacteria, Patescibacteria, Myxococcota, and Bacteroidota were all the top 4 common dominant bacterial phyla in rhizospheres of MS and MT (Figure 2a). However, the relative abundances of Proteobacteria and Bacteroidota in rhizospheres of MT were significantly higher than those of MS; conversely, the abundance of Chloroflexi in rhizospheres of MS was significantly higher than that of MT.

At the genus level, 23 and 20 dominant bacterial genera could be found in rhizospheres of MS and MT, respectively. Among them, *Acidibacter*, *Bryobacter*, *Streptomyces*, *Acidicaldus*, *Rhodanobacter*, *Bradyrhizobium*, *Jatrophihabitans*, *Occallatibacte*, *Bacillus*, *Acidothermus*, and *Sphingomonas* were the common dominant bacterial genera in rhizospheres of MS and MT (Figure 2b). Meanwhile, the abundances of *Streptomyces*, *Acidibacter*, *Rhodanobacter*, and *norank_f__Micropepsaceae* in rhizospheres of MT were significantly higher than those of MS. Conversely, the abundances of *norank__f__JG30-KF-AS9* and *norank__f__norank__o___Gaiellales* in rhizospheres of MT were significantly lower than those of MS. Moreover, *norank_f__norank_o__Subgroup_13*, *norank_f__norank_o__Subgroup_2*, and *norank_f__norank_o__Acidobacteriales* were the special dominant soil bacterial genera in rhizospheres of MS. By contrast, specific dominant soil bacterial genera could not be detected in rhizosphere of MT.

Additionally, a total of 4 dominant fungal phyla, i.e., Basidiomycota, Ascomycota, Mortierellomycota, and unclassified -K- fungi could be detected in rhizospheres of MS and MT (Figure 2c). At the genus level, 19 and 21 dominant fungal genera could be detected in rhizospheres of MS and MT, respectively. Among them, *Gymnopilus*, *Penicillium*, *Aspergillus*, *Talaromyces*, and *Derxomyces* were the common dominant fungal genera in rhizospheres of MS and MT (Figure 2d). Moreover, the abundances of *Gymnopilus*, *Penicillium*, *Derxomyces*, and *unclassified_c_Agaricomycetes* were significantly higher in rhizospheres of MT than those of MS. Furthermore, *Mycothermus*, *Zopfiella*, *Cladorrhinum*, and *unclassified_c_Agaricomycetes* were the special dominant fungal genera in rhizospheres of MT. By contrast, *Gibellulopsis* and *Pseudallescheria* were the unique dominant fungal genera in the rhizospheres of MS.

**Figure 3:**
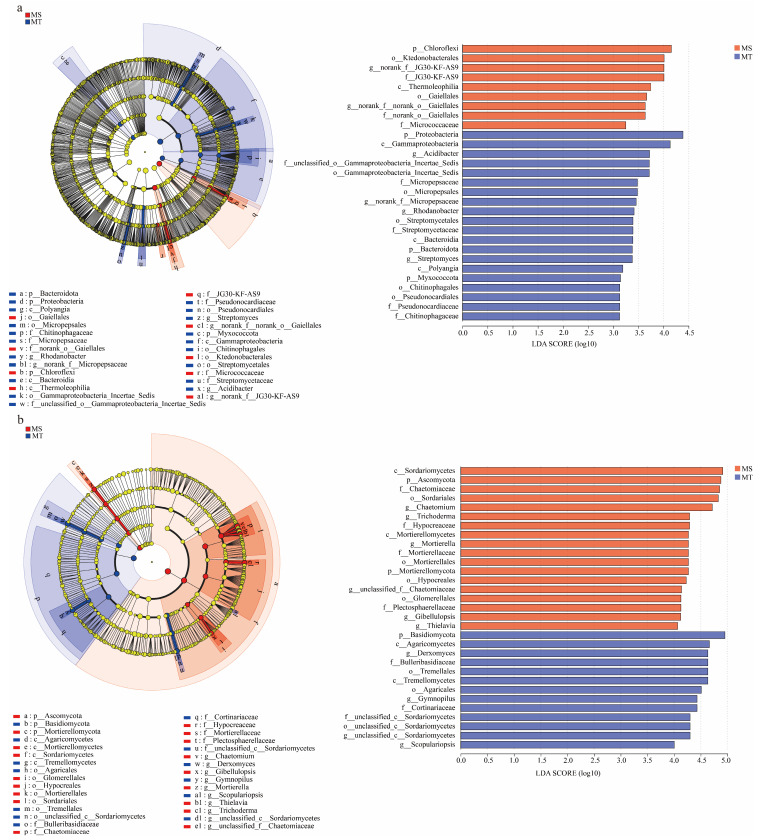
LEfSE analysis of soil bacteria (**a**) and fungi (**b**) in rhizospheres between MT and MS varieties.

As shown in Figure 3a, 9 and 20 soil bacterial clades in rhizospheres showed differences (*p* < 0.05, LDA threshold of 3.0) between MS and MT, respectively. Among them, at the phylum level, Chloroflexi was significantly clustered in rhizospheres of MS. In contrast, Proteobacteria, Bacteroidota, and Myxococcota were significantly enriched in rhizospheres of MT. At the genus level, *norank__f__JG30-KF-AS9* and *norank__f__norank__o___Gaiellales* were significantly enriched in the MS rhizosphere. By contrast, *Acidibacter*, *norank__f__Micropepsaceae*, *Rhodanobacter*, and *Streptomyces* were significantly aggregated in the MT rhizosphere.

Also, as shown in Figure 3b, 18 and 13 soil fungal clades were found in rhizospheres of MS and MT (*p* < 0.05, LDA threshold of 4.0), respectively. Among them, at the phylum level, Ascomycota and Mortierellomycota were significantly enriched in rhizospheres of MS, and Basidiomycota was significantly clustered in rhizospheres of MT. At the genus level, *Chaetomium*, *Trichoderma*, *Mortierella*, unclassified_f__*Chaetomiaceae*, *Gibellulopsis*, and *Thielavia* were significantly enriched in rhizospheres of MS, and *Derxomyces*, *Gymnopilus*, unclassified_c__*Sordariomycetes*, and *Scopulariopsis* were significantly aggregated in rhizospheres of MT.

### 3.3. Venn Diagram Analysis Soil Microorganisms in Rhizosphere Between Wilt-Resistant and Susceptible Melon Varieties

**Figure 4:**
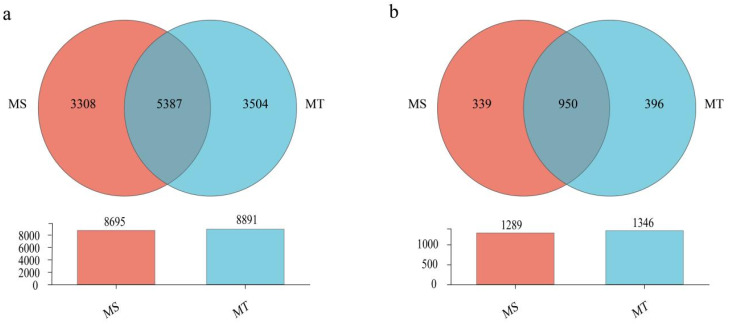
Venn diagrams of the soil-dominant bacteria (**a**) and soil fungi (**b**) in rhizospheres between wilt-resistant (MT) and susceptible melons (MS) varieties at the OTU levels.

As shown in Figure 4a, 8891 and 8695 soil bacterial OTUs were found in rhizospheres of MT and MS, respectively. Among them, 5387 common soil-bacterial OTUs and 3504 and 3308 unique bacterial OTUs were detected in rhizospheres of MT and MS, respectively. Meanwhile, 1346 and 1289 fungal OTUs were also found in rhizospheres of MT and MS, respectively. Moreover, 950 common soil-fungi OTUs with 396 and 339 unique fungal OTUs were detected in rhizospheres of MT and MS, respectively (Figure 4b).

### 3.4. Co-Generation Network Structure of Soil Microbial Communities in Rhizospheres of Wilt-Resistant (MT) and Susceptible Melons Varieties (MS)

**Figure 5:**
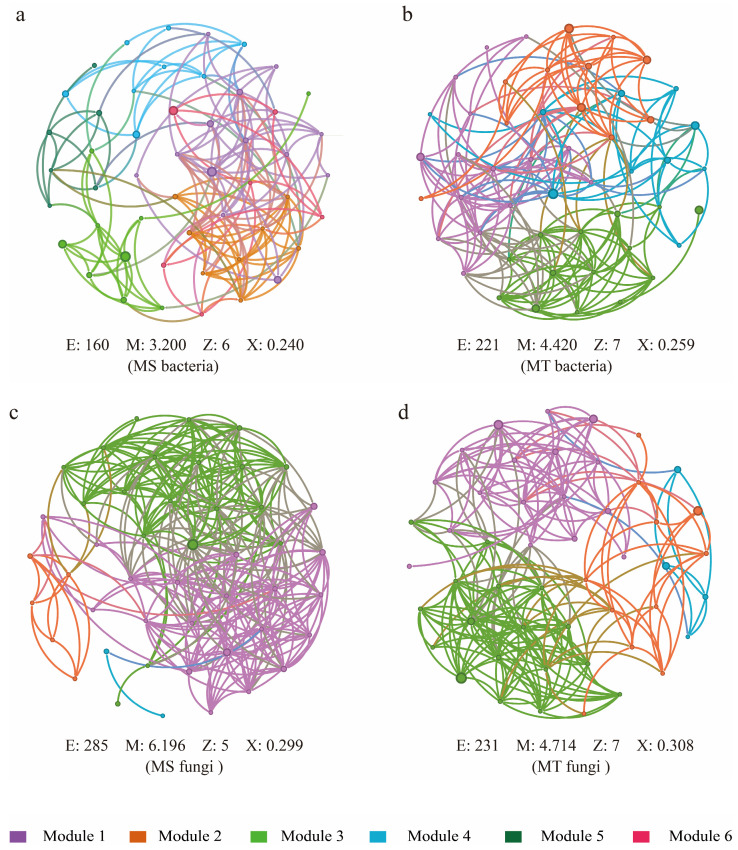
Co-generation network of the bacteria of MS (**a**) and MT (**b**); co-generation network of the fungi of MS (**c**) and MT (**d**); each node represents a single genus, and different colors represent different modules. E, M, Z, and X represent the number of edges, average degree, network diameter, and average clustering coefficient, respectively.

To investigate the interspecific relationships of soil microorganisms in rhizospheres of different resistant melon varieties, a univariate correlation network analysis (Spearman correlation coefficient ≥0.5, *p* < 0.05) was also performed. The results showed that more network diameters and average clustering coefficients could be detected in rhizospheres of MT than those of susceptible melon varieties (MS) (Figure 5). Also, 4 and 6 modules of soil bacterial networks could be found in the rhizospheres of MT and MS, respectively. Meanwhile, 51.25% positive and 48.75% negative connections could be found in rhizospheres of MS (Figure 5a). In contrast, 53.85% positive and 46.15% negative connections could be detected in rhizospheres of MT (Figure 5b). Additionally, in soil fungal network analyses, 55.09% positive and 44.91% negative connections were found in rhizospheres of MS (Figure 5c). By contrast, 60.17% positive and 39.83% negative connections were detected in rhizospheres of MT (Figure 5d). All the above results indicate that soil microbial networks, particularly soil bacterial communities in rhizospheres of resistant melons, are higher in stability than those of susceptible melon varieties.

### 3.5. Functional Predictions of Soil Microbial Communities in Rhizospheres of Wilt-Resistant (MT) and Susceptible Melons Varieties (MS)

**Figure 6:**
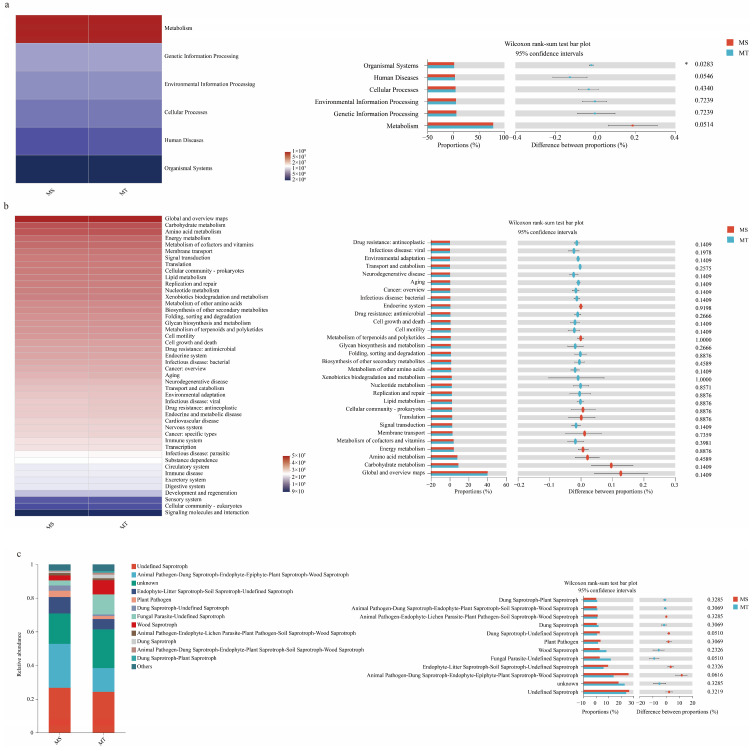
Functional predictions of soil bacterial and fungal communities in rhizospheres of MT and MS melon varieties. Compositional variability test for bacterial (**a**,**b**) and fungal (**c**) communities. * *p* ≤ 0.05.

Based on the KEGG (Kyoto Encyclopedia of Genes and Genomes) database, 6 metabolic pathways of soil bacterial communities, i.e., metabolism, genetic information processing, environmental information processing, cellular processes, human diseases, and organismal systems, were detected in rhizospheres between MS and MT (Figure 6a).

Also, 46 soil bacterial metabolic pathways, such as global and overview maps, carbohydrate metabolism, amino acid metabolism, energy metabolism, metabolism of cofactors and vitamins, and another 46 subfunctions, were found in rhizospheres of MS and MT (Figure 6b). However, there was no significant difference in rhizospheres between MS and MT varieties.

Additionally, as shown in Figure 6c, 12 functional categories of soil fungal communities, such as Animal Pathogen-Dung Saprotroph-Endophyte-Epiphyte-Plant Saprotroph-Wood Saprotroph and Dung Saprotroph-Undefined Saprotroph in rhizospheres of MT, were significantly higher than those of MS. In contrast, Parasite-Undefined Saprotroph fungi were significantly lower in rhizospheres of MT than those of MS.

## 4. Discussion

Wilt is a devastating disease in melon production, and its pathogens can survive in soil for a long time. Traditional agricultural measures are slow, time-consuming, and laborious, and chemical pesticides are toxic and harmful to the environment, so biological control has become an important way of integrated pest management [45]. Currently, there are limited resources of antagonistic microorganisms that can effectively control melon wilt. Previous studies have found that soil-beneficial microorganisms in rhizospheres of melon could promote plant growth and reduce wilt by producing iron carriers and antibiotic substances and secretion of exogenous compounds [46].

In our study, we also found that the abundances of Proteobacteria, Bacteroidota, *Streptomyces*, and *Acidibacter* enriched in rhizospheres of MT were comparable to those of MS. Microorganisms in rhizospheres play a crucial role in plant health against soil-borne diseases [47]. Proteobacteria and Bacteroidota played an important role in biological nitrogen fixation [48], promoting soil organic phosphorus mineralization [49]. Meanwhile, *Streptomyces* not only produces various bioactive substances such as antibiotics, herbicides, insecticides, and plant growth promoters [50,51], but also inhibits Fusarium through resource competition [52]. Also, *Acidibacter* is strongly and positively correlated with soil CH_4_ oxidation potential [53]. *Rhodanobacte* could produce indole-3-acetic acid and ammonia, dissolve phosphate, and form siderophore, which was an important growth-promoting bacterium [54]. Additionally, LEfSE analysis found that the rhizosphere of MS was enriched with two unclassified bacterial genera, *norank__f__JG30-KF-AS9* and *Norank_f_norank_o_Gaiellales*. Although the roles of these genera are not clearly reported, studies have shown that the relative abundance of *norank__f__JG30-KF-AS9* is negatively correlated with root exudates related to grapevine plant resistance to replant disease. It is speculated that *norank__f__JG30-KF-AS9* has a negative effect on plant disease resistance [55]. Potassium (K) is an essential nutrient for plants, and most bacterial and fungal diseases decrease with increased K nutrition [56]. Sufficient K concentrations can increase the concentration of polyphenols in plants, which play a key role in defense mechanisms [57]. Gu et al. also found that *Norank_f_norank_o_Gaiellales* was significantly negatively correlated with K [58]. In addition, wilt-resistant varieties not only detected antagonist pathogenic bacteria such as Proteobacteria and *Streptomyces* in the rhizosphere soil, but also had a more stable structure of the rhizospheres’ bacterial flora, compared with wilt-susceptible varieties.

In the fungal community structure, we found that the fungal richness index in the MT rhizosphere was significantly higher than in MS. MT enriched more fungi such as *Penicillium*, *Derxomyces*, *Aspergillus*, and *Talaromyces*. Previous studies also reported that *Derxomyces*, *Penicillium*, and *Aspergillus* in soil were significantly negatively correlated with banana wilt pathogens [59]. Al-Hawash et al. found that Aspergillus can effectively reduce soil pollution by secreting various soil-active enzymes that decompose harmful substances, thereby creating more favorable environmental conditions for root growth [60]. Inoculation with *Talaromyces* could promote the lignin biosynthesis pathway in reducing the incidence of wilt disease [61]. Meanwhile, in comparison with MS, we found that *Mycothermus*, *Zopfiella*, *Cladorrhinum*, and *unclassified_c_Agaricomycetes* were the dominant fungal genera in rhizospheres of MT. *Mycothermus* has been reported as an important indicator of wilt-diseased soils [62], and *Zopfiella* possessed antimicrobial activity against a wide range of pathogens [63,64]. Also, some species of *Cladorrhinum* had biocontrol potential in promoting plant growth and producing phytase [65,66]. *Unclassified_c_Agaricomycetes* had been reported to be associated with better organic matter utilization [67]. On the contrary, the abundance of *Trichoderma* in rhizospheres of MS was significantly higher than that of MT. Moreover, *Gibellulopsis* and *Pseudallescheria* were the specific dominant fungal genera in rhizospheres of MS, which were known plant pathogens that can cause various plant diseases [68,69,70].

Studies have shown that a high abundance of beneficial microbial groups with functional traits in the rhizosphere can strengthen the first line of defense for plants against pathogens [43]. In our study, the rhizosphere of fusarium wilt-resistant melons was enriched with beneficial functional microbes such as Streptomyces and Zopfiella, which may be one of the important reasons why resistant melon varieties are less susceptible to pathogen infection. Our findings are also similar to those of fusarium disease-resistant tobacco varieties, which could provide a better micro-ecological environment in reducing the spread of fusarium wilt [71].

## 5. Conclusions

Although soil bacterial diversity and richness were not significantly different in rhizospheres between wilt-resistant (MT) and susceptible (MS) melon varieties, the soil fungal richness in rhizospheres of wilt-resistant melon varieties (MT) was significantly higher than that of MS. Additionally, in comparison with wilt-susceptible melon varieties (MS), soil bacterial compositions, such as Proteobacteria, Bacteroidota, *Streptomyces*, *Acidibacter*, etc., and the soil fungal compositions, such as *Penicillium*, *Derxomyces*, *Aspergillus*, and *Talaromyces*, were enriched, and *Trichoderma*, *Gibellulopsis*, and *Pseudallescheria* decreased in rhizospheres of wilt-resistant melon varieties (MT). Moreover, *Mycothermus*, *Zopfiella*, and *Cladorrhinum* were the unique soil-dominant fungal genera in rhizospheres of MT.

All the above results indicated that the bacterial communities, such as Proteobacteria, Bacteroidota, *Streptomyces*, *Acidibacter*, etc., and the soil fungal communities, such as *Penicillium*, *Derxomyces*, *Aspergillus*, *Talaromyces Mycothermus*, *Zopfiella*, and *Cladorrhinum*, could be considered as the potential soil bio-control microorganisms for preventing melon wilt.

## Figures and Tables

**Table 1:** Sequence type and primer sequences.

Sequence Type	Primer Name	Primer Sequence	Length Sequencing
Bacterial 16S rRNA	338F	5′-ACTCCTACGGGAGGCAGCAG-3′	416 bp
	806R	5′-GGACTACHVGGGTWTCTAAT-3′	
Fungal ITS	ITS1F	5′-CTTGGTCATTTAGAGGAAGTAA-3′	242 bp
	ITS2R	5′-GCTGCGTTCTTCATCGATGC-3′	

**Table 2:** Soil microbial diversity in rhizospheres between wilt-resistant and susceptible melon varieties.

	Treatments	Shannon	Simpson	ACE	Chao 1	Coverage
Rhizosphere bacteria	CK	5.67 ± 0.07 b	0.01 ± 0.00 a	2434.56 ± 71.04 b	2349.97 ± 43.24 b	0.99
	MS	6.12 ± 0.01 a	0.01 ± 0.00 b	3393.99 ± 41.51 a	3270.81 ± 58.11 a	0.98
	MT	6.35 ± 0.14 a	0.01 ± 0.00 b	3711.33 ± 124.54 a	3560.2 ± 129 a	0.98
Rhizosphere fungi	CK	3.96 ± 0.18 a	0.04 ± 0.01 a	485.53 ± 50.73 b	482.69 ± 52.18 b	1.00
	MS	3.83 ± 0.24 a	0.05 ± 0.01 a	566.39 ± 45.16 b	558.45 ± 45.06 b	1.00
	MT	4.02 ± 0.23 a	0.04 ± 0.01 a	628.69 ± 54.95 a	621.48 ± 49.00 a	1.00

Notes: Data shown are mean ± standard deviation. Different letters in the same column indicate significant differences (two-tailed Duncan’s test) between treatments at *p* < 0.05.

## Data Availability

The data presented in this study are openly available in [National Center for Biotechnology Information] at [https://www.ncbi.nlm.nih.gov/, accessed on 15 March 2024], reference number [PRJNA1088444, PRJNA1088463].

## References

[1] Soriano-Martın M.L., Porras-Piedra A., Porras-Soriano A. (2006). Use of microwaves in the prevention of *Fusarium oxysporum* f. sp. melonis infection during the commercial production of melon plantlets. Crop Prot..

[2] Imazaki I., Kadota I. (2019). Control of *fusarium* wilt of melon by combined treatment with biocontrol, plant-activating, and soil-alkalizing agents. J. Gen. Plant Pathol..

[3] Zhao Q., Dong C., Yang X., Mei X., Ran W., Shen Q., Xu Y. (2011). Biocontrol of *fusarium* wilt disease for *Cucumis* melo melon using bio-organic fertilizer. Appl. Soil Ecol..

[4] Oumouloud A., Arnedo-Andrés M.S., González-Torres R., Alvarez J.M. (2009). Morphological and molecular characterization of melon accessions resistant to *fusarium* wilts. Euphytica.

[5] Fang S., Tang L., Chen S., Gu G., Chen Y. (2011). Influence of crop rotation on tobacco bacterial wilt number and pothogenesy. Chin. J. Eco-Agric..

[6] Everts K.L., Egel D.S., Langston D., Zhou X. (2014). Chemical management of *fusarium* wilt of watermelon. Crop Prot..

[7] Kanaan H., Medina S., Raviv M. (2017). The effects of Soil solarization and compost on soil suppressiveness against *Fusarium oxysporum* f. sp. Melonis. Compos. Sci. Util..

[8] Gava C.A.T., Pinto J.M. (2016). Biocontrol of melon wilt caused by *fusarium oxysporum Schlect* f. sp. melonis using seed treatment with *Trichoderma* spp. and liquid compost. Biol. Control.

[9] Mehta C.M., Palni U., Franke-Whittle I.H., Sharma A.K. (2014). Compost: Its role, mechanism and impact on reducing soil-borne plant diseases. Waste Manag..

[10] King S.R., Davis A.R., Liu W., Levi A. (2008). Grafting for disease resistance. HortScience.

[11] Karimi H., Mahdavi S., Asgari Lajayer B., Moghiseh E., Rajput VDMinkina T., Astatkie T. (2022). Insights on the bioremediation technologies for pesticide-contaminated soils. Environ. Geochem. Health.

[12] Tamietti G., Valentino D. (2006). Soil solarization as an ecological method for the control of *fusarium* wilt of melon in Italy. Crop Prot..

[13] Ku C., Guo L., Cheng F., Kuo C., Karim A.F., Hardiansyah M.Y., Chang Y., Chen Y., Chung Y., Cheng C. (2024). The levels of pattern-triggered immunity in the root and stembase of tomato cultivars positively correlate with the resistance to *Ralstonia solanacearum*. Bot. Stud..

[14] Cal D.A., Pascua S., Larena I., Melgarejo P. (1995). Biological control of *Fusarium oxysporum* f. sp. lycopersici. Plant Pathol..

[15] Larkin R.P., Fravel D.R. (1998). Efficacy of various fungal and bacterial biocontrol organisms for control of Fusarium wilt of tomato. Plant Dis..

[16] Ulloa-Ogaz A.L., Muñoz-Castellanos L.N., Nevárez-Moorillón G.V. (2015). Biocontrol of phytopathogens: Antibiotic production as mechanism of control. The Battle Against Microbial Pathogens: Basic Science, Technological Advances and Educational Programs.

[17] Muslim A., Horinouchi H., Hyakumachi M. (2003). Biological control of fusarium wilt of tomato with hypovirulent binucleate Rhizoctonia in greenhouse conditions. Mycoscience.

[18] Gong M., Wang J.D., Zhang J., Yang H., Lu X.F., Pei Y., Cheng J.Q. (2006). Study of the antifungal ability of *Bacillus subtilis* strain PY-1 in vitro and identification of its antifungal substance (iturin A). Acta Biochim. Biophys. Sin..

[19] Zhang Y., Wang X., Liang S., Shi Y., Chen X., Liu J., Wang A. (2021). Fermentation Optimization, Fungistatic Effects and Tomato Growth Promotion of Four Biocontrol Bacterial Strains. Agriculture.

[20] Meng J., Zan F., Liu Z., Zhang Y., Qin C., Hao L., Wang Z., Wang L., Liu D., Liang S. (2024). Genomics Analysis Reveals the Potential Biocontrol Mechanism of *Pseudomonas aeruginosa* QY43 against *Fusarium pseudograminearum*. Fungi.

[21] Sun H., Jiang S., Jiang C., Wu C., Gao M. (2021). A review of root exudates and rhizosphere microbiome for crop production. Environ. Sci. Pollut. Res..

[22] Feng H., Fu R., Luo J., Hou X., Gao K., Su L., Xu Y., Miao Y., Liu Y., Xu Z. (2023). Listening to plant’s Esperanto via root exudates: Reprogramming the functional expression of plant growth-promoting rhizobacteria. New Phytol..

[23] Zhao Y., Lu Q., Wei Y., Cui H., Zhang X., Wang X., Shan S., Wei Z. (2016). Effect of actinobacteria agent inoculation methods on cellulose degradation during composting based on redundancy analysis. Bioresour. Technol..

[24] Suleman M., Yasmin S., Rasul M., Yahya M., Atta B.M., Mirza M.S. (2018). Phosphate solubilizing bacteria with glucose dehydrogenase gene for phosphorus uptake and beneficial effects on wheat. PLoS ONE.

[25] Zhou J., Fan X., Li J., Wang X., Yuan Z. (2021). Isolation and identification of naphthalene degrading bacteria and their degradation characteristics under rainwater environment in heavily polluted areas. J. Environ. Sci. Health Part A.

[26] Sokolova M.G., Akimova G.P., Vaishlya O.B. (2011). Effect of phytohormones synthesized by rhizosphere bacteria on plants. Appl. Biochem. Microbiol..

[27] Raaijmakers J.M., Paulitz T.C., Steinberg C., Alabouvette C., Moënne-Loccoz Y. (2009). The rhizosphere: A playground and battlefield for soilborne pathogens and beneficial microorganisms. Plant Soil.

[28] Shi H., Xu P., Wu S., Yu W., Cheng Y., Chen Z., Yang X., Yu X., Li B., Ding A. (2022). Scientific Reports Analysis of rhizosphere bacterial communities of tobacco resistant and non-resistant to bacterial wilt in different regions. Sci. Rep..

[29] Berendsen R., Pieterse C.M.J., Bakker P.A.H.M. (2012). The rhizosphere microbiome and plant health. Trends Plant Sci..

[30] Van Elsas J.D., Chiurazzi M., Mallon C.A., Elhottova D., Kristufek V., Salles J.F. (2011). Microbial diversity determines the invasion of soil by a bacterial pathogen. Proc. Natl. Acad. Sci. USA.

[31] Bulgarelli D., Garrido-Oter R., Münch Philipp C.M., Weiman A., Dröge J., Pan Y., McHardy A.C., Schulze-Lefert P. (2015). Structure and Function of the Bacterial Root Microbiota in Wild and Domesticated Barley. Cell Host Microbe.

[32] Ajilogba C.F., Olanrewaju O.S., Babalola O.O. (2022). Plant Growth Stage Drives the Temporal and Spatial Dynamics of the Bacterial Microbiome in the Rhizosphere of *Vigna subterranea*. Front. Microbiol..

[33] Rayanoothala P., Hasibul Alam S., Mahapatra S., Gafur A., Antonius S. (2023). Rhizosphere microorganisms for climate resilient and sustainable crop production. Gesunde Pflanz..

[34] Kwak M., Kong H.G., Choi K., Kwon S., Song J.Y., Lee J., Lee P.A., Choi S.Y., Seo M., Lee H.J. (2018). Rhizosphere microbiome structure alters to enable wilt resistance in tomato. Nat. Biotechnol..

[35] Chen S., Chen Z., Lin X., Zhou X., Yang S., Tan H. (2023). Why different sugarcane cultivars show different resistant abilities to smut?. BMC Plant Biol..

[36] Chikh-Rouhou H., González-Torres R., Oumouloud A., Alvarez J. (2011). Inheritance of race 1.2 fusarium wilt resistance in four melon cultivars. Euphytica.

[37] Joobeur T., King J.J., Nolin S.J., Thomas C.E., Dean R.A. (2004). The fusarium wilt resistance locus Fom-2 of melon contains a single resistance gene with complex features. Plant J..

[38] Tezuka T., Waki K., Kuzuya M., Ishikawa T., Takatsu Y., Miyagi M. (2011). Development of new DNA markers linked to the fusarium wilt resistance locus Fom-1 in melon. Plant Breed..

[39] Spanic V., Viljevac Vuletic M., Abicic I. (2017). MarcekT Early response of wheat antioxidant system with special reference to fusarium head blight stress. Plant Physiol. Biochem..

[40] Sadeghpour N., Asadi-Gharneh H.A., Nasr-Esfahani M., Khankahdani H.H., Golabad M. (2022). Antioxidant enzymes associated with resistance to *Fusarium oxysporum* f. sp. melonis race 1.2 in melon. Physiol. Mol. Plant Pathol..

[41] Ma Y., Wu F., Liu S. (2008). Pathohistological and histological studies on wilt-susceptible cucumber varieties. Plant Prot..

[42] Chen MShang F., Jiang J., Zhou Y., An G., Wang P., Miao Y., Song C. (2004). Cytological studies on wilt resistance in watermelon. J. Sichuan Univ. Nat. Sci. Ed..

[43] Feng Z., Liang Q., Yao Q., Bai Y., Zhu H. (2024). The role of the rhizobiome recruited by root exudates in plant disease resistance: Current status and future directions. Environ. Microbiome.

[44] Riley D., Barber S.A. (1970). Salt accumulation at the Soybean (*Glycine* Max. (L.) Merr.) root-soil interface1. Soil Sci. Soc. Am. J..

[45] Raymaekers K., Ponet L., Holtappels D., Berckmans B., Cammue B.P.A. (2020). Screening for novel biocontrol agents applicable in plant disease management—A review. Biol. Control.

[46] Registeri R., Taghavi S.M., Banihashemi Z. (2012). Effect of Root Colonizing Bacteria on Plant growth and *fusarium* wilt in Cucumis melo. J. Agric. Sci. Technol..

[47] Chang X., Wei D., Zeng Y., Zhao X., Hu Y., Wu X., Song C., Gong G., Chen H., Yang C. (2022). Maize-soybean relay strip intercropping reshapes the rhizosphere bacterial community and recruits beneficial bacteria to suppress *fusarium* root rot of soybean. Front. Microbiol..

[48] Guo J., Li Q., Gao Q., Shen F., Yang Y., Zhang X., Luo H. (2023). Comparative study on the treatment of swine wastewater by VFCW-MFC and VFCW: Pollutants removal, electricity generation, microorganism community. J. Environ. Manag..

[49] Bi Q.F., Li K.J., Zheng B.X., Liu X.P., Li H.Z., Jin B.J., Ding K., Yang X.R., Lin X.Y., Zhu Y.G. (2020). Partial replacement of inorganic phosphorus (P) by organic manure reshapes phosphate mobilizing bacterial community and promotes P bioavailability in a paddy soil. Sci. Total Environ..

[50] Olsthoorn-Tieleman L.N., Palstra R.T.S., Wezel G.P., Bibb M.J., Pleij C.W.A. (2007). Elongation Factor Tu3 (EF-Tu3) from the kirromycin producer *Streptomyces ramocissimus* is pesistant to three classes of EF-Tu-Specific inhibitors. J. Bacteriol..

[51] Ikeda H., Omura S. (1997). Avermectin biosynthesis. Chem. Rev..

[52] Essarioui A., LeBlanc N., Kistler H.C., Kinke L. (2017). Plant community richness mediates inhibitory interactions and resource competition between Streptomyces and Fusarium Populations in the rhizosphere. Microb. Ecol..

[53] Yang H., Lee J., Cho K.S. (2023). Dynamics of Functional Genes and Bacterial Community during Bioremediation of Diesel-Contaminated Soil Amended with Compost. J. Microbiol. Biotechnol..

[54] Woo H., Kim I., Chhetri G., Park S., Lee H., Yook S., Seo T. (2024). Two novel bacterial species, *Rhodanobacter lycopersici* sp. nov. and *Rhodanobacter geophilus* sp. nov., isolated from the rhizosphere of solanum *lycopersicum* with plant growth-promoting traits. Microorganisms.

[55] Zhang J., Liu Q., Li K., Ma L. (2022). Peanut root exudates suppress *Fusarium solani* and modulate the microbial community structure of rhizosphere in grape replant soil. Horticulturae.

[56] Ortel C.C., Roberts T.L., Rupe J.C. (2024). A review of the interaction between potassium nutrition and plant disease control. Agrosystems Geosci. Environ..

[57] Tripathi R., Tewari R., Singh K.P., Keswani C., Minkina T., Srivastava A.K., Corato U.D., Sansinenea E. (2022). Plant mineral nutrition and disease resistance: A significant linkage for sustainable crop protection. Front. Plant Sci..

[58] Gu Y., Zhang H., Liang X.Y., Fu R., Li M., Chen C. (2022). Effect of different biochar particle sizes together with bio-organic fertilizer on rhizosphere soil microecological environment on saline–alkali land. Front. Environ. Sci..

[59] Liu M. (2024). Study on the Process of Building Microbiota and Its Driving Mechanism in Green Manure Set in Banana Plantations. Master’s Thesis.

[60] Al-Hawash A.B., Zhang J., Li S., Liu J., Ghalib H.B., Zhang X., Ma F. (2018). Biodegradation of n-hexadecane by *Aspergillus* sp. RFC-1 and its mechanism. Ecotoxicol. Environ. Saf..

[61] Tian Y., Fu X., Yan X., Li X., Peng H., Gao K. (2022). The control efficacy and mechanism of *Talaromyces purpurogenus* on Fusarium wilt of bitter gourd. Biol. Control Theory Appl. Pest Manag..

[62] Yuan J., Wen T., Zhang H., Zhao M., Penton C.R., Thomashow L.S., Shen Q. (2020). Predicting disease occurrence with high accuracy based on soil macroecological patterns of *fusarium* wilt. ISME J..

[63] Yi X.W., He J., Sun L.T., Liu J., Wang G., Feng T. (2021). 3-Decalinoyltetramic acids from kiwi-associated fungus *Zopfiella* sp. and their antibacterial activity against *Pseudomonas syringae*. RSC Adv..

[64] Zhang J., He J., Li Z., Feng T., Liu J. (2021). Zopfiellasins A-D, two pairs of fpimeric cytochalasins from kiwi-associated fungus *Zopfiella* sp. and their antibacterial assessment. Molecules.

[65] Barrera V.A., Martin M.E., Aulicino M., Martínez S., Chiessa G., Saparrat M.C.N., Gasoni A.L. (2019). Carbon-substrate utilization profiles by *Cladorrhinum* (Ascomycota). Rev. Argent. Microbiol..

[66] Carmarán C.C., Berretta M., Martínez S., Barrera V., Munaut F., Gasoni L. (2015). Species diversity of *Cladorrhinum* in Argentina and description of a new species, *Cladorrhinum australe*. Mycol. Prog..

[67] Zhang L. (2022). Research on Rhizosphere Microbiota of Artificially Restored Vegetation in the Three Gorges Reservoir Fallout Zone. Master’s Thesis.

[68] Peng J., Dong B., Wang D., Zhao X., Meng H., Zhou H. (2022). Analysis of differential metabolites of sunflower induced by virulent *Verticillium* dahlia V33 and hypovirulent *Gibellulopsis nigrescens* Vn-1. J. Phytopathol..

[69] Li T., Wang M., Cui R., Li B., Wu T., Liu Y., Geng G., Xu Y., Wang Y. (2023). Waterlogging stress alters the structure of sugar beet rhizosphere microbial community structure and recruiting potentially beneficial bacterial. Ecotoxicol. Environ. Saf..

[70] Rodrigo R.P., De M.J.V., Vila T.V.M., Fonseca B.B., Alves V., Frases S., Rozental S., Barreto-Bergter E. (2017). Biofilm Formation by *Pseudallescheria/Scedosporium* Species: A Comparative Study. Front. Microbiol..

[71] Xia H., Shen J., Riaz M., Yang H., Dong Q., Zu C., Yu F., Yan Y., Li J., Liu B. (2024). Microbial Regulatory Mechanisms of Disease-resistant Tobacco Varieties in The Prevention and Control of Bacterial Wilt Disease. Appl. Soil Ecol..

